# Response of alfalfa growth to arbuscular mycorrhizal fungi and phosphate-solubilizing bacteria under different phosphorus application levels

**DOI:** 10.1186/s13568-020-01137-w

**Published:** 2020-11-03

**Authors:** Junying Liu, Xuanshuai Liu, Qianbing Zhang, Shengyi Li, Yanliang Sun, Weihua Lu, Chunhui Ma

**Affiliations:** College of Animal Science & Technology, Shihezi University, Shihezi, 832003 Xinjiang China

**Keywords:** Alfalfa, AMF, Phosphate-solubilizing bacteria, Growth traits, Nutritional quality

## Abstract

Alfalfa (*Medicago sativa* L.) is an important forage legume in farming and animal husbandry systems. This study assessed the effects of arbuscular mycorrhizal fungi (AMF) and phosphate-solubilizing bacteria (PSB) on alfalfa growth under different phosphorus application levels. In this experiment, a complete randomized block design was used. The following four bacterial applications were used: inoculation of *Funneliformis mosseae* (Fm), inoculation of *Bacillus megaterium* (Bm), inoculation of mixed species (Fm × Bm) and noninoculation treatment (CK). Phosphorus (P) treatment was applied at the following four levels: 0 mg kg^−1^ (P_0_), 50 mg kg^−1^ (P_1_), 100 mg kg^−1^ (P_2_) and 150 mg P kg^−1^ (P_3_). The results showed that with the increase in phosphorus application, each index increased first and then decreased. The J_2_ treatment was significantly greater than the J_0_ treatment (*P *< 0.05) under the same bacterial treatment. In each cropping period the difference in each index to alfalfa was extremely significant under J, P treatment and J × P interactive treatment (*P *< 0.01). The indexes were compared by membership function. The priority order was as follows: J_3_P_2_ > J_1_P_2_ > J_3_P_1_ treatment. Therefore, when phosphorus was applied at 100 mg kg^−1^, the mixed inoculation of Fm × Bm was optimal, benefitting mycorrhiza growth and the production performance of alfalfa.

## Introduction

Alfalfa (*Medicago sativa* L.), as a perennial legume forage, is a high-quality protein feed for livestock, and it is an essential cultivated forage in the arid and semiarid areas of western China (Gu et al. 2018). Phosphorus is an indispensable nutrient element in plants, and it is one of the main limiting factors for crop yield increase. At present, many studies have shown that phosphate fertilizer is a significant way to improve crop phosphorus nutrition and increase crop yield (Gill 2019). After phosphate fertilizer is applied to soil, the growth of alfalfa branches is significantly improved, thereby increasing the regeneration rate of alfalfa plants and the hay yield of alfalfa (Berg et al. 2018; Zhang et al. 2020a). Phosphorus application plays a vital role in promoting the further increase of alfalfa hay production.

Arbuscular mycorrhizal fungi (AMF) are widely distributed and are one of the microorganisms most closely related to plants in the soil. AMF plays a critical role in the uptake of plant nutrients in the natural environment, and they promote the absorption of phosphorus nutrients and enhance the resistance of plants to biological and abiotic stresses (Jeffries et al. 2003; Li et al. 2011). Studies have demonstrated that AMF form a symbiotic relationship with more than 80% of land plants (Smith and Read 2008). AMF form cysts on twigs and in the endothelial cells of plant roots, thereby forming mutually beneficial symbiotic plants. This symbiosis helps plants to improve the rhizosphere microenvironment, increase the absorption of mineral elements by the host, improve stress resistance, improve disease resistance and promote plant growth (Li et al. 2019). At the same time, plants provide the required carbon source and energy for mycorrhizal fungi, and they form a massive extraroot hypha network in the soil. AMF expand the range of plant root absorption and enhance the plant’s uptake of nitrogen, phosphorus and water to improve the nutritional status of host plants, thereby promoting plant growth (Gyuricza et al. 2010; Victor et al. 2013). Studies have shown that inoculation with AMF increases the chlorophyll content in the leaves, enhances the photosynthetic efficiency and reduces the toxic effects caused by adversity stress by changing the plant’s osmotic adjustment ability and related antioxidant enzyme activities (Goicoechea et al. 2014). Therefore, inoculation of AMF has a significant promoting effect on plants.

Similar to AMF, phosphate-solubilizing bacteria (PSB) are important microorganisms in the soil for phosphorus uptake by plants. PSB secrete organic acids and enzymes, and they convert organic phosphorus and insoluble phosphorus into useful phosphorus that can be absorbed and utilized by plants, which improves the utilization efficiency of phosphorus in the soil, thereby promoting the growth of crops (Louche et al. 2010). Studies have shown that mixed inoculation of PSB and AMF significantly increases crop yields (Khan et al. 2007). The content of soluble sugar, as the primary index of carbohydrate storage and transportation in alfalfa, is of considerable significance to the study of alfalfa growth and nutrition. At present, many types of research have focused on the improvement of mycorrhizae infection rate and soluble sugar content (Wang et al. 2012; Karasawa et al. 2012). However, there have been few studies to investigate the effects of mixed inoculation and interaction of bacteria and phosphorus on alfalfa growth as well as relationship between indicatorst. In particular, the contribution rate of bacteria and phosphorus to alfalfa plant growth is rarely reported. Therefore, this study surveyed the effects of inoculation of AMF and PSB on the alfalfa mycorrhizae infection rate, growth traits, nutritional quality and soluble sugar content at different phosphorus application levels to clarify the relationship between indicators and the effect of bacterial phosphorus interaction on alfalfa growth. The optimal bacterial phosphorus coupling model suitable for alfalfa with high quality and high yield was selected.

## Materials and methods

### Experimental materials

For the tested strain, AM fungus Funneliformis mosseae (Fm) was selected. The inoculant was rhizosphere soil, including host plant root, mycorrhizal fungal spore and ectomycorrhizal mycelium. The density of the spore was 25–35 g, which was provided by the Qingdao Agricultural Mycorrhizae Research Institute of China.

For the phosphate-solubilizing bacteria, Bacillus megaterium (Bm) were purchased from the Agricultural Culture Collection of China (ACCC; No. 10011). The host plant alfalfa variety tested was WL354HQ.

### Experimental design

The experiment adopted a two-factor random block design with two factors of bacterial application and phosphorus application. The following four bacterial treatments were used: single inoculation of *Funneliformis mosseae* (Fm), single inoculation of *Bacillus megaterium* (Bm), inoculation of mixed strains (Fm × Bm) and noninoculated bacteria (CK), which were labeled as J_1_, J_2_, J_3_, and J_0_, respectively. In the single inoculation treatment group, 10 g of bacteria was used in each pot. In the mixed inoculation treatment, 5 g of each bacterial strain was applied in each pot (approximately 8500 inoculation potential units). The noninoculated (J_0_) treatment strains were subjected to sterilization. The noninoculated (J_0_) treatment strains were subjected to sterilization. The following four levels of phosphorus treatment were used, and each treatment was repeated 6 times: 0 mg kg^−1^ (P_0_), 50 mg kg^−1^ (P_1_), 100 mg kg^−1^ (P_2_) and 150 mg kg^−1^ (P_3_).

The experiment was performed at the Shihezi University Experimental Base (44°18′N, 86°03′E) from May to October 2019. The pot experiment was conducted with a nutrient bowl with a top diameter of 23 cm, a bottom diameter of 15 cm and a height of 16 cm. The test soil was derived from the test field of Shihezi University (44°26′N, 85°95′E). The soil was sterilized in a high-pressure steam sterilizer at 121 °C for 2 h and then air-dried for use, and each pot was filled with 3 kg to destroy the fungus in the dried soil. To promote root colonization of alfalfa, we inoculated *Funneliformis mosseae* (Fm) 5 cm below the soil surface in the pot. Full and uniformly sized alfalfa seeds were selected and disinfected with 10% H_2_O_2_ for 10 min, repeatedly washed with distilled water and seeded on May 1, 2019 with ten seeds per pot. Equivalent water supply was provided for every pot after sowing, and seedlings were interplanted when seedlings grew to the three-leaf stage. Each cup retained five alfalfa seedlings with the same growth trend. Each treatment was replicated six times, and the pots were randomly placed. The phosphate fertilizer used was pandemonium phosphate (containing 52% of P and 11% of N). Because pandemonium phosphate contains N, urea (containing 46% of N) was added to maintain the same N content in each treatment. All fertilizers were implemented in two batches on June 18, 2019 and September 19, 2019, and the method of fertilization was to supply by water drops. Alfalfa was harvested in two crops throughout the year at the initial flowering stage (flowering 5–10%), and the remaining stubble height was 2 cm. The specific cutting time was August 2, 2019 and October 12, 2019.

### Measurement index and method

#### Aboveground biomass

Each pot was considered as a unit, and three pots of alfalfa plants with consistent growth were selected. The alfalfa plants in the sample plot (cut to 2 cm) were cut with scissors and weighed, and the yield of fresh alfalfa forage was recorded three times for every treatment. A soil shaking method was used to remove the underground part of the alfalfa plant. The root system was collected, rinsed and weighed, and the fresh weight was recorded. The samples were first oven-dried at 105 °C for 30 min and then at 65 °C to a constant mass. The moisture content was measured and converted into alfalfa aboveground biomass (g pot^−1^) and underground biomass (g pot^−1^) using the following formulas:

1 $${\text{A}} = {\text{FY}} \times (1 - {\text{MC}})$$

2 $${\text{U}} = {\text{FR}} \times (1 - {\text{MC}})$$

where A is aboveground biomass of alfalfa (g pot^−1^); FY is fresh yield of alfalfa (g pot^−1^); MC is the moisture content; U is underground biomass of alfalfa (g pot^−1^); and FR is fresh root of alfalfa (g pot^−1^).

#### Plant height and stem diameter

While measuring aboveground biomass, alfalfa plants representing the growth of the plot were selected, and the absolute height of the alfalfa plants was measured with a steel tape measure. The average value (cm) of the absolute height was reported. A Vernier caliper with an accuracy of 0.02 mm was used to measure the alfalfa at 5 cm from the ground stem diameter (mm).

#### Nutritional quality

The crude protein content (CP) was determined using the Kjeldahl method (Bradstreet 1965). The acid detergent fiber (ADF) and neutral detergent fiber (NDF) contents were determined according to the Van Soest method (Van Soest et al. 1991).

#### Mycorrhizal infection rate

After harvesting, the mycorrhizae infection rate of alfalfa was measured, and 0.5 g of fresh alfalfa root segments was weighed. The root segments were reduced into 0.5–1.0 cm sections and put into test tubes. Next, 20 mL of 5% KOH was added, and the tubes were kept in a 90 °C water bath for 20–60 min. The root system was then rinsed three times with tap water and soaked in 20 mL of 2% HCl solution for 5 min. The acid solution was removed, and 20 mL of 0.01% acid fuchsin lactic acid glycerin staining solution was then added. The tubes were then placed back in a 90 °C water bath for 20–60 min, and 20 mL of lactic acid was then added to separate the color. Samples were then examined using a stereo microscope. Fifteen root segments were randomly selected and fixed on one slide, and two slices were performed, resulting in 30 stained plant fibrous root segments. These root segments were examined under a microscope and counted (Talaat and Shawky 2014). The mycorrhizal infection rate (MIR) was calculated using the following formula:

3 $${\text{MIR}} = {\text{NS}}/{\text{TNS}} \times 100$$

where MIR is mycorrhizal infection rate in roots (%); NS is number of mycorrhizal segments; and TNS is total number of root segments.

#### Chlorophyll content and soluble sugar content

After the aboveground biomass of alfalfa was measured, fresh leaves of alfalfa plants were harvested, and chlorophyll content was determined by the acetone method (Cui and Yang 2011). The soluble sugar content was determined by the anthrone method (Feil and Lunn 2018). Each index was repeated three times, and the average value (mg kg^−1^) was obtained.

### Data processing and analysis

Microsoft Excel 2010 was used for data processing. SPSS 18.0 (SPSS Inc., Chicago, IL, USA) and DPS 8.0 software (Data Processing System, China) were used for statistical analyses. Two-way ANOVA was used to analyze the interaction of J, P and J × P, and Duncan’s method was used for multiple comparisons. Data were plotted with Origin 8.0 software (OriginLab and OriginPro, USA) and expressed as the mean ± standard error.

Pearson’s correlation coefficient measures the degree of correlation between two variables, and it is a value between 1 and − 1 as follows: 1 detects an entirely positive relationship of variables; 0 indicates no correlation; and − 1 indicates an entirely negative correlation. The correlation among mycorrhizae infection rate, growth traits and soluble sugar content of alfalfa was analyzed by Pearson’s correlation analysis.

The subordinate function evaluation method was used to comprehensively evaluate the optimal treatment using the following formulas:

4 $${\text{UX}}( + ) = ({\text{Xij}} - {\text{Ximin}})/({\text{Ximax}} - {\text{Ximin}})$$

5 $${\text{UX}}( - ) = 1 - {\text{UX}}( + )$$

where X is the measured value of each index of the sample; UX(+) is the positive correlation low function value of each index; and UX(−) is the negative correlation low function value of each index.

## Results

### Effect of vaccination on the mycorrhizae infection rate of alfalfa under different phosphorus applications

**Fig. 1:**
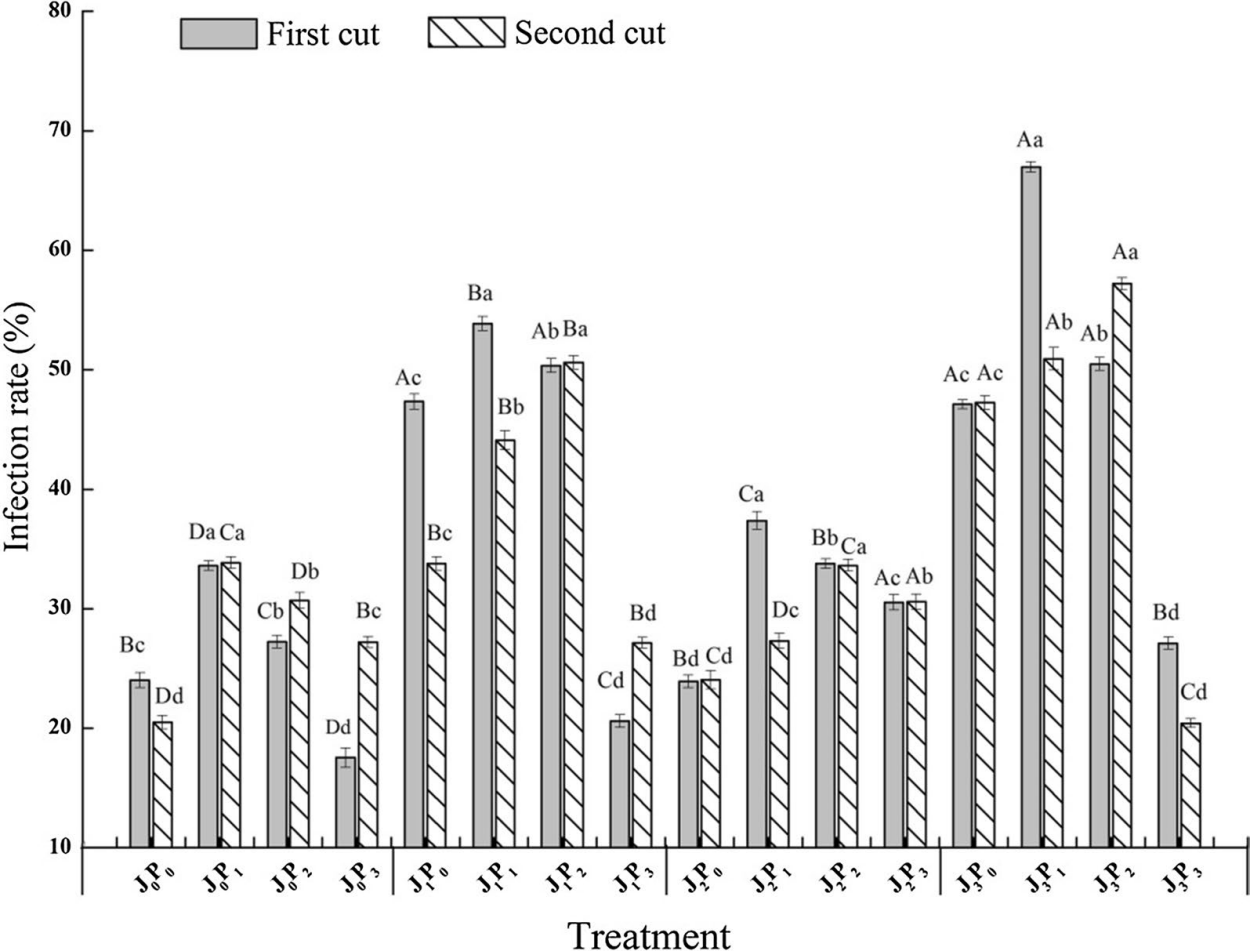
Mycorrhizal infection rate of alfalfa inoculated with AMF and PSB under different phosphorus application conditions. Different capital letters indicated significant difference in different bacteria treatments under the same phosphorus application conditions(*P *< 0.05), differences small letters mean significant difference under the same bacteria treatments(*P *< 0.05). P_0_, P_1_, P_2_, and P_3_ represent 0 mg kg^−1^, 50 mg kg^−1^, 100 mg kg^−1^, and 150 mg P kg^−1^, respectively. J_0_, J_1_, J_2_, and J_3_ represent CK, Fm, Bm, and Fm × Bm, respectively

The mycorrhizae infection rate of alfalfa increased first and then decreased with the increase of phosphorus application rate under the same treatment conditions (Fig. 1). The first crop reached the highest infection rate under the P_1_ treatment, and the P_1_ treatment had a significantly higher infection rate than that under the P_0_ and P_2_ treatments (*P* < 0.05). The second crop reached the highest infection rate under the P_2_ treatment, except for the J_0_ treatment, and the P_2_ treatment had a significantly higher infection rate than that under the P_0_, P_1_ and P_3_ treatments (*P* < 0.05). Phosphorus application treatment resulted in a higher infection rate than without phosphorus application under the J_2_ treatment, and the P_1_ treatment resulted in a higher infection rate than the P_2_ and P_3_ treatments (*P *< 0.05). Both single inoculation (J_1_ or J_2_) and mixed inoculation (J_3_) treatments had significantly higher infection rates than those without vaccination (J_0_) (*P *< 0.05) under the same phosphorus application treatment.

**Fig. 2:**
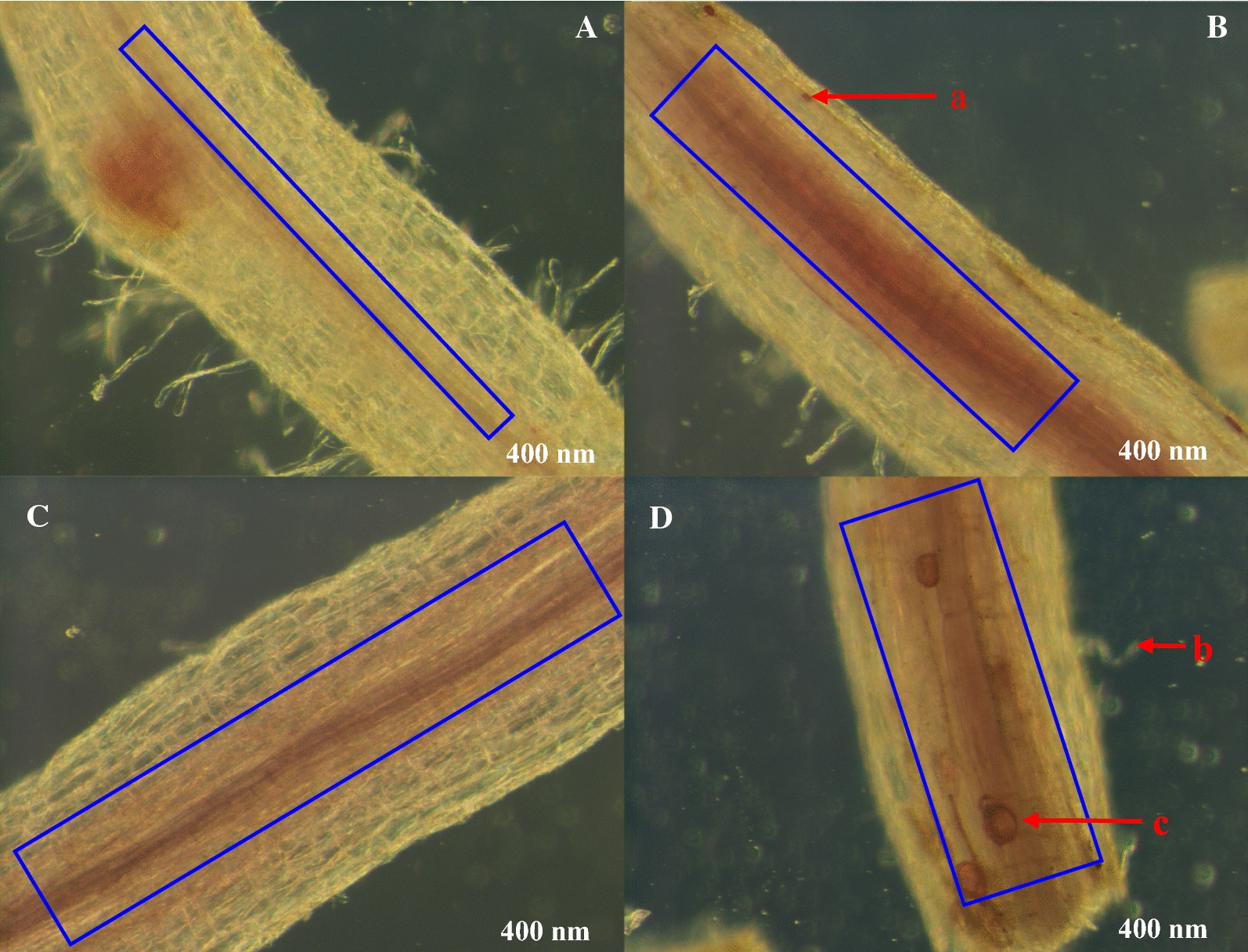
The degree of mycorrhizal infection under different treatments under stereomicroscope. **A**, **B**: Single inoculation of *Funneliformis mosseae* (Fm), **C** Single inoculation of *Bacillus megaterium* (Bm), **D** Mixed Inoculation Fm × Bm, a: Root spores; b: Mycelium; c: Vesicle. The blue box indicates the degree of mycorrhizal infection

The degree of alfalfa mycorrhizae infection varied under different treatments (Fig. 2). Compared to the untreated (CK) treatment, the alfalfa mycorrhizae infection rate was higher under the single (Fm or Bm) and mixed (Fm × Bm) treatments (blue box area in Fig. 2). Among the treatments, alfalfa mycorrhizae infection was the highest under the mixed inoculation treatment (Fig. 2D). The order of mycorrhizae infection degree under different treatments was as follows: Fm × Bm (Fig. 2D) > Bm (Fig. 2C) > Fm (Fig. 2B) > CK (Fig. 2A). As the degree of infection increased, a small intraroot spore was found in the single inoculation treatment (Fm) (Fig. 2B-a). At the same time, hyphae (Fig. 2D-b) and vesicles (Fig. 2D-c) began to appear in the alfalfa mycorrhizas under the mixed inoculation (Fm × Bm) treatment.

### Effect of vaccination on the aerial growth of alfalfa under different phosphorus applications

The aboveground biomass, plant height and stem diameter of alfalfa increased first and then decreased with the increase of phosphorus application (Table 1). The aerial growth after all treatments was significantly greater in the P_2_ treatment than in P_0_ treatment, except that the aboveground biomass of the first crop reached the highest value under the P_1_ treatment (*P *< 0.05). Compared to the noninoculated treatment, all treatments, except the P_1_ treatment under the J_3_ condition (i.e., J_3_P_1_), reached the maximum value under the P_2_ treatment under the J_3_ condition (i.e., J_3_P_2_). The aboveground biomass, plant height and stem diameter of alfalfa under different inoculation treatments were greater in the first crop than in the second crop. The aboveground biomass, plant height and stem diameter of alfalfa under the J, P and J × P treatments were significantly different in each cultivation (*P *< 0.01).

**Table 1:** The growth of alfalfa under different bacterial treatments

Treatments	Aboveground biomass (g pot^−1^)		Plant height (cm)		Stem diameter (mm)	
	First cut	Second cut	First cut	Second cut	First cut	Second cut
J_0_P_0_	13.13 ± 0.38^Dd^	11.47 ± 0.23^Dd^	30.33 ± 0.59^Dd^	23.60 ± 0.53^Bd^	1.93 ± 0.04^Bb^	1.60 ± 0.07^Cb^
J_0_P_1_	25.36 ± 0.57^Ca^	11.99 ± 0.49^Dc^	34.53 ± 0.43^Cc^	24.67 ± 0.29^Bc^	2.19 ± 0.02^Aa^	2.32 ± 0.06^Aa^
J_0_P_2_	23.04 ± 0.62^Cb^	17.67 ± 0.37 ^Da^	40.25 ± 0.57^Ca^	32.37 ± 0.40 ^Da^	2.25 ± 0.05^Aa^	2.23 ± 0.01^Ca^
J_0_P_3_	18.98 ± 0.34^Cc^	15.36 ± 0.26^Db^	36.72 ± 0.28^Bb^	26.27 ± 0.10^Cb^	2.00 ± 0.01^Bb^	2.26 ± 0.06^Aa^
J_1_P_0_	18.24 ± 0.23^Cd^	20.83 ± 0.78^Ac^	34.15 ± 0.50^Bd^	32.50 ± 0.50^Ad^	2.16 ± 0.02^Ab^	1.73 ± 0.04^Bc^
J_1_P_1_	26.37 ± 0.59^Ba^	27.18 ± 0.36^Aa^	36.52 ± 0.20^Bc^	34.00 ± 0.50^Ac^	2.18 ± 0.01^Ab^	2.29 ± 0.02^Aa^
J_1_P_2_	23.50 ± 0.41^Bb^	26.96 ± 0.37^Ba^	42.38 ± 0.31^Ba^	40.00 ± 0.50^Ba^	2.27 ± 0.04^Aa^	2.17 ± 0.03^Cb^
J_1_P_3_	22.50 ± 0.45^Ac^	26.47 ± 0.43^Ab^	38.90 ± 0.33^Ab^	35.5 ± 0.44^Ab^	2.24 ± 0.04^Aab^	2.10 ± 0.04^Bb^
J_2_P_0_	23.26 ± 0.60^Bc^	15.17 ± 0.45^Bd^	35.26 ± 0.29^Ac^	32.53 ± 0.50^Ab^	1.90 ± 0.01^Bb^	1.65 ± 0.04^Bc^
J_2_P_1_	24.49 ± 0.14^Db^	17.75 ± 0.31^Cc^	37.58 ± 0.48^Aa^	34.50 ± 0.44^Aa^	1.93 ± 0.01^Bab^	2.11 ± 0.04^Bb^
J_2_P_2_	25.40 ± 0.87^Aa^	24.98 ± 0.12^Aa^	36.04 ± 0.19^Db^	35.13 ± 0.60^Ca^	2.01 ± 0.01^Ca^	2.46 ± 0.01^Ba^
J_2_P_3_	19.74 ± 0.67^Bd^	22.14 ± 0.38^Cb^	35.47 ± 0.24^Cbc^	30.37 ± 0.21^Bc^	1.87 ± 0.02^Cb^	2.08 ± 0.06^Bb^
J_3_P_0_	25.09 ± 0.38^Ab^	14.05 ± 0.17^Cc^	31.35 ± 0.23^Cd^	32.5 ± 0.50^Ad^	1.73 ± 0.04^Cb^	2.08 ± 0.04^Ab^
J_3_P_1_	27.03 ± 0.61^Aa^	23.17 ± 0.35^Bb^	37.32 ± 0.48^Ac^	34.5 ± 0.50^Ac^	1.80 ± 0.06^Cb^	2.16 ± 0.08^Bb^
J_3_P_2_	21.64 ± 0.26^Dc^	28.88 ± 0.15^Ca^	46.98 ± 0.54^Aa^	44.47 ± 0.46^Aa^	2.14 ± 0.07^Ba^	2.57 ± 0.05^Aa^
J_3_P_3_	19.87 ± 0.43^Bd^	23.38 ± 0.32^Bb^	39.53 ± 0.42^Ab^	35.87 ± 0.32^Ab^	1.78 ± 0.06^Db^	2.15 ± 0.04^Bb^
J	**	**	**	**	**	**
P	**	**	**	**	**	**
J × P	**	**	**	**	**	**

Different capital letters in the same column indicated significant difference in different bacteria treatments under the same phosphorus application conditions (*P *< 0.05), differences small letters in the same column mean significant difference under the same bacteria application conditions (*P *< 0.05). ** indicates significant difference extremely (*P *< 0.01)

P_0_, P_1_, P_2_, and P_3_ represent 0 mg kg^−1^, 50 mg kg^−1^, 100 mg kg^−1^, and 150 mg P kg^−1^, respectively. J_0_, J_1_, J_2_, and J_3_ represent CK, Fm, Bm, and Fm × Bm, respectively

### Effect of vaccination on the CP content of alfalfa under different phosphorus applications

**Fig. 3:**
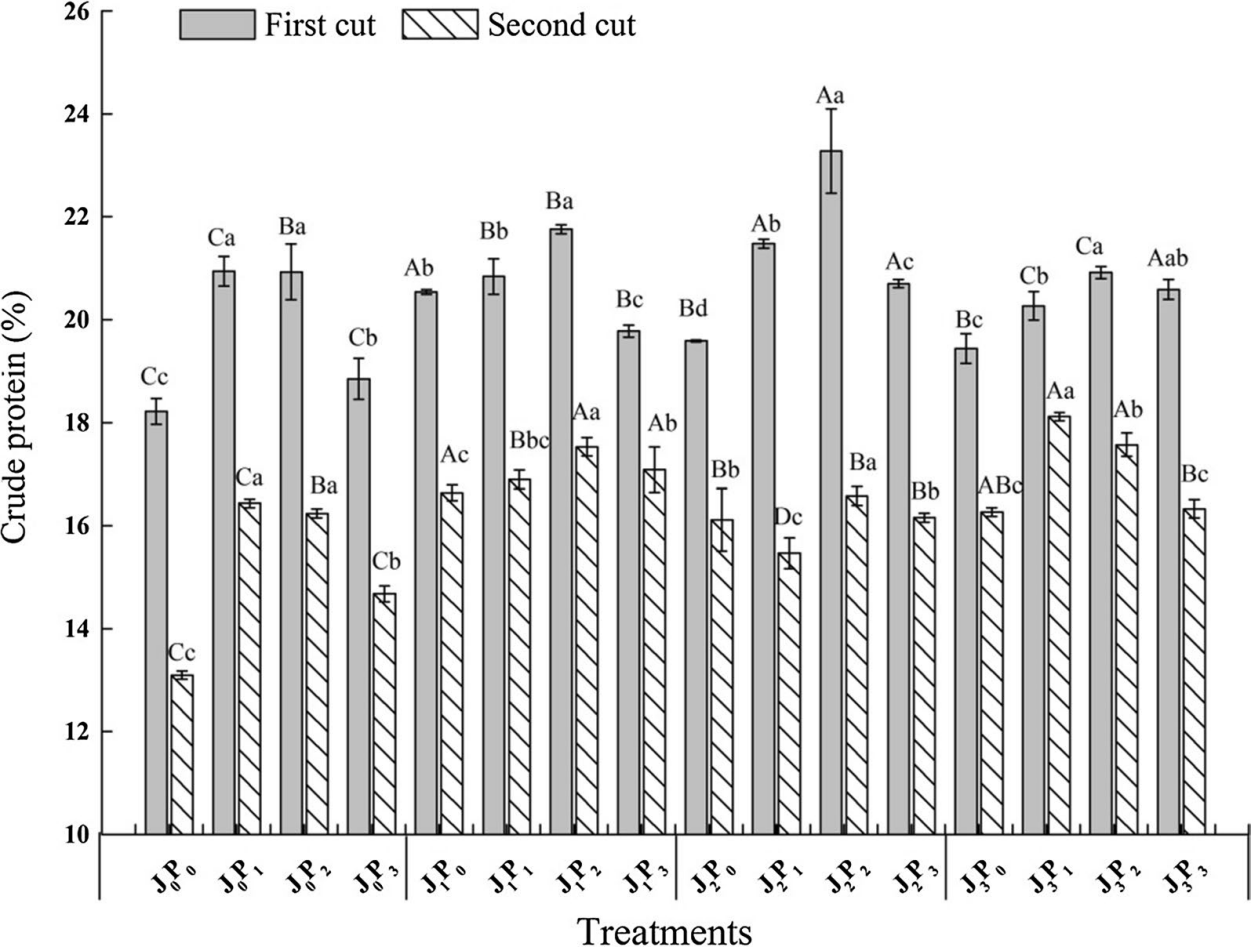
Crude protein content of alfalfa inoculated with AMF and PSB under different phosphorus application conditions

The CP content of alfalfa increased first and then decreased with the increase of phosphorus application under the same bacterial application conditions (Fig. 3). The CP content of alfalfa reached the highest value under the P_2_ treatment with the J_0_ condition, except for the second crop P_3_ treatment, and the P_2_ treatment was significantly greater than the P_0_ treatment (*P *< 0.05). The difference in CP content between the P_1_ treatment and the P_2_ treatment was not significant (*P *> 0.05). Under the J_1_ and J_2_ conditions, the P_2_ treatment resulted in significantly higher CP content than that of the P_0_, P_1_ and P_3_ treatments (*P *< 0.05). The CP content reached the highest value under the P_2_ treatment with the J_3_ condition, except for the second crop P_1_ treatment, and there was no significant difference between the P_2_ and P_3_ treatments (*P *> 0.05). The CP content after treatment with J_2_ in the first crop was markedly higher than that of alfalfa treated with J_0_ and J_3_ under the same phosphorus application conditions (*P *< 0.05). The CP content of alfalfa treated with J_2_ in the second crop was considerably higher than that after treatment with J_0_, J_2_ and J_3_ (*P *< 0.05). Under the P_1_ condition, the CP content of alfalfa treated with J_2_ in the first crop was significantly greater than that after treatment with J_0_ (*P *< 0.05). The CP content of alfalfa treated with J_3_ in the second crop was substantially higher than that of alfalfa treated with J_0_ and J_2_ (*P *< 0.05). Under the conditions of P_0_ and P_2_, the CP contents of alfalfa treated with J_1_ and J_3_ were significantly greater than that of alfalfa treated with J_0_ (*P *< 0.05), and there was no significant difference in CP contents among the J_1_, J_2_ and J_3_ treatments (*P *> 0.05). The alfalfa CP content after the J_1_ treatment was significantly greater than that after the J_0_ treatment under the P_3_ condition (*P *< 0.05), and there was no significant difference between the J_2_ and J_3_ treatments (*P *> 0.05).

### Effect of vaccination on the alfalfa NDF and ADF contents under different phosphorus applications

The NDF and ADF of alfalfa first increased and then decreased with increasing phosphorus application under the same bacterial application (Table 2). The NDF and ADF contents after all treatments were significantly greater than those with P_3_ treatment (*P *< 0.05). The NDF content after treatment with P_1_ was significantly greater than that after treatment with P_0_ with the J_0_ and J_3_ conditions (*P *< 0.05), and the NDF content after treatment with P_2_ was the lowest with the J_2_ condition. The NDF and ADF contents of alfalfa after all treatments were lower than that of the J_0_ treatment under the constant phosphorus application condition, except for the NDF content after the J_3_ treatment in the first crop, which was markedly greater than that of J_0_ treatment under the constant phosphorus application condition (*P *< 0.05). The NDF and ADF contents in alfalfa were higher in the first crop than in the second crop under different inoculation treatment conditions. The NDF and ADF contents of alfalfa were significantly different under the J, P and J × P treatments in each plant (*P *< 0.01).

**Table 2:** Neutral detergent fiber and acid detergent fiber of alfalfa under different treatments (%)

Treatments	NDF (%)		ADF (%)	
	First cut	Second cut	First cut	Second cut
J_0_P_0_	54.49 ± 0.87^Bc^	48.62 ± 0.42^Ac^	41.98 ± 0.84^Cc^	37.97 ± 0.34^Ac^
J_0_P_1_	58.83 ± 0.40^Aa^	51.05 ± 0.57^Bb^	46.82 ± 0.44^Ab^	39.45 ± 0.49^Ab^
J_0_P_2_	57.47 ± 0.46^Cb^	46.89 ± 0.04^Bd^	48.49 ± 0.48^Aa^	41.76 ± 0.23^Aa^
J_0_P_3_	57.36 ± 0.45^Ab^	54.98 ± 0.79^Aa^	46.10 ± 0.33^Ab^	41.01 ± 0.44^Aa^
J_1_P_0_	54.09 ± 0.58^Bc^	45.89 ± 0.65^Bc^	45.57 ± 0.54^Ab^	36.67 ± 0.37^Bb^
J_1_P_1_	54.61 ± 0.20^Cc^	52.40 ± 0.30^Aa^	45.70 ± 0.87^Bb^	38.88 ± 0.40^Aa^
J_1_P_2_	59.54 ± 0.46^Ba^	50.98 ± 0.16^Ab^	47.43 ± 0.66^Ba^	38.58 ± 0.43^Ba^
J_1_P_3_	55.60 ± 0.47^Bb^	45.80 ± 0.29^Bc^	46.73 ± 0.82^Aa^	35.70 ± 0.61^Cc^
J_2_P_0_	51.20 ± 0.49^Cc^	45.75 ± 0.96^Bc^	42.01 ± 0.66^Cb^	38.30 ± 0.79^Aa^
J_2_P_1_	56.76 ± 0.67^Ba^	49.45 ± 0.28^Ca^	42.63 ± 0.63^Cab^	37.94 ± 0.20^Ba^
J_2_P_2_	54.34 ± 0.34^Dd^	47.81 ± 0.47^Bb^	43.56 ± 0.49 ^Da^	38.49 ± 0.72^Ba^
J_2_P_3_	54.41 ± 0.37^Cb^	45.25 ± 0.68^Bc^	41.89 ± 0.47^Bb^	36.19 ± 0.31^Cb^
J_3_P_0_	57.98 ± 0.20^Ac^	42.48 ± 0.54^Cc^	44.54 ± 0.46^Bb^	35.89 ± 0.23^Bc^
J_3_P_1_	59.09 ± 0.29^Ab^	49.38 ± 0.10^Ca^	46.10 ± 0.17^ABa^	39.65 ± 0.63^Aa^
J_3_P_2_	61.71 ± 0.55^Aa^	43.97 ± 0.65^Cb^	46.06 ± 0.28^Ca^	36.45 ± 0.89^Cbc^
J_3_P_3_	53.98 ± 0.30^Cd^	43.63 ± 0.69^Cb^	41.81 ± 0.76^Bc^	37.11 ± 0.72^Bb^
J	**	**	**	**
P	**	**	**	**
J × P	**	**	**	**

Different capital letters in the same column indicated significant difference in different bacteria treatments under the same phosphorus application conditions (*P *< 0.05), differences small letters in the same column mean significant difference under the same bacteria application conditions (*P *< 0.05). ** indicates significant difference extremely (*P *< 0.01)

P_0_, P_1_, P_2_, and P_3_ represent 0 mg kg^−1^, 50 mg kg^−1^, 100 mg kg^−1^, and 150 mg P kg^−1^, respectively. J_0_, J_1_, J_2_, and J_3_ represent CK, Fm, Bm, and Fm × Bm, respectively

### Effect of vaccination on chlorophyll content and leaf soluble sugar content of alfalfa under different phosphorus applications

The chlorophyll content and leaf soluble sugar content of alfalfa first increased and then decreased with increasing phosphorus application under the same phosphorus application, except for the second crop under the J_2_ and J_3_ conditions (Table 3). The chlorophyll content and soluble sugar content in the first crop was significantly higher after the P_1_ treatment than after the other treatments under the J_0_ condition (*P *< 0.05). In the second crop, the chlorophyll content and soluble sugar content reached the highest values under the P_2_ treatment. The chlorophyll content after the P_2_ treatment was significantly greater than that after the other treatments under the J_1_ condition (*P *< 0.05). The soluble sugar content in the first crop was significantly higher in the P_3_ treatment that after the other treatments (*P *< 0.05), and the soluble sugar content in the second crop was higher after the P_1_ treatment than after the P_0_ treatment with the J_1_ condition (*P *< 0.05). Both indexes of the first crop were significantly higher after the P_2_ treatment compared to the other treatments with the J_2_ condition (*P *< 0.05), and the chlorophyll content of the second crop was higher after P_1_ treatment than after the other treatments (*P *< 0.05). The chlorophyll content in the first crop was higher after the P_1_ treatment than after the other treatments (*P *< 0.05), and the chlorophyll content in the second crop and the soluble sugar content in the first crop were significantly higher after the P_2_ treatment than after the other treatments with the J_3_ condition (*P *< 0.05). The soluble sugar content in the second crop was considerably higher after the P_3_ treatment than after the P_0_ treatment with the J_3_ condition (*P *< 0.05). The inoculation treatment was markedly greater than the noninoculation treatment under the same phosphorus application conditions (*P *< 0.05). In each crop, the chlorophyll content and soluble sugar content were significantly different under the J, P and J × P treatments (*P *< 0.01).

**Table 3:** Chlorophyll content and Content of soluble suger of alfalfa under different treatments (mg kg^−1^)

Treatments	Chlorophyll content (mg kg^−1^)		Content of soluble suger (mg kg^−1^)	
	First cut	Second cut	First cut	Second cut
J_0_P_0_	2.34 ± 0.010^Dd^	1.80 ± 0.008^Cd^	1.26 ± 0.008^Dd^	1.93 ± 0.007^Dd^
J_0_P_1_	2.91 ± 0.006^Ca^	2.04 ± 0.002^Cc^	1.68 ± 0.003 ^Da^	2.07 ± 0.010^Dc^
J_0_P_2_	2.45 ± 0.009^Db^	2.74 ± 0.006^Ca^	1.51 ± 0.004^Db^	2.97 ± 0.006^Ca^
J_0_P_3_	2.37 ± 0.007^Dc^	2.59 ± 0.003^Bb^	1.43 ± 0.012^Dc^	2.50 ± 0.004^Db^
J_1_P_0_	2.39 ± 0.023^Cd^	2.41 ± 0.005^Bc^	1.62 ± 0.002^Bd^	2.44 ± 0.007^Bd^
J_1_P_1_	3.15 ± 0.003^Bb^	2.55 ± 0.009^Bb^	1.76 ± 0.008^Cc^	3.12 ± 0.006^Aa^
J_1_P_2_	3.35 ± 0.007^Aa^	2.87 ± 0.005^Ba^	2.11 ± 0.009^Bb^	3.04 ± 0.004^Bb^
J_1_P_3_	3.09 ± 0.008^Ac^	1.92 ± 0.010^Dd^	2.45 ± 0.006^Aa^	2.68 ± 0.008^Cc^
J_2_P_0_	2.45 ± 0.023^Bd^	1.62 ± 0.010^Dd^	1.44 ± 0.007^Cd^	2.04 ± 0.007^Cd^
J_2_P_1_	2.64 ± 0.006^Db^	2.54 ± 0.010^Ba^	2.16 ± 0.004^Ba^	2.48 ± 0.005^Cc^
J_2_P_2_	2.82 ± 0.004^Ca^	2.51 ± 0.010^Db^	1.59 ± 0.006^Cb^	2.90 ± 0.008^Db^
J_2_P_3_	2.48 ± 0.005^Cc^	2.46 ± 0.010^Cc^	1.53 ± 0.001^Cc^	3.87 ± 0.007^Aa^
J_3_P_0_	2.76 ± 0.004^Ad^	2.92 ± 0.010^Ac^	2.07 ± 0.005^Ad^	2.45 ± 0.014^Ad^
J_3_P_1_	3.38 ± 0.009^Aa^	3.10 ± 0.004^Ab^	3.55 ± 0.005^Ab^	2.66 ± 0.007^Bc^
J_3_P_2_	3.15 ± 0.005^Bb^	3.39 ± 0.004^Aa^	4.15 ± 0.009^Aa^	3.06 ± 0.011^Ab^
J_3_P_3_	2.83 ± 0.005^Bc^	2.84 ± 0.006^Ad^	2.12 ± 0.010^Bc^	3.36 ± 0.011^Ba^
J	**	**	**	**
P	*	**	**	**
J × P	**	**	**	**

Different capital letters in the same column indicated significant difference in different bacteria treatments under the same phosphorus application conditions (*P *< 0.05), differences small letters in the same column mean significant difference under the same bacteria application conditions (*P *< 0.05). ** indicates significant difference extremely (*P *< 0.01)

P_0_, P_1_, P_2_, and P_3_ represent 0 mg kg^−1^, 50 mg kg^−1^, 100 mg kg^−1^, and 150 mg P kg^−1^, respectively. J_0_, J_1_, J_2_, and J_3_ represent CK, Fm, Bm, and Fm × Bm, respectively

### Correlation analysis of alfalfa indexes under different treatments

The aboveground biomass of alfalfa was positively correlated with plant height and crude protein content (*P *< 0.01), and the aboveground biomass was positively correlated with mycorrhizae infection rate, stem diameter and chlorophyll content (*P *< 0.05) (Table 4). The mycorrhizae infection rate was positively correlated with chlorophyll content (*P *< 0.01). The mycorrhizae infection rate was positively correlated with crude protein content (*P *< 0.05). Alfalfa plant height was positively correlated with stem diameter and crude protein content (*P *< 0.05). Stem diameter was positively correlated with crude protein content, NDF and ADF (*P *< 0.05). The crude protein content was positively correlated with chlorophyll content and soluble sugar content (*P *< 0.05). There was a significant positive correlation between NDF and ADF contents (*P *< 0.01).

**Table 4:** The correlation analysis of each index of alfalfa under different bacterial treatments

Index	Aboveground biomass (g pot^−1^)	Infection rate (%)	Plant height (cm)	Stem diameter (mm)	Crude protein content (%)	NDF (%)	ADF (%)	Chlorophyll content (mg kg^−1^)
Infection rate (%)	0.604*							
Plant height (cm)	0.736**	0.463						
Stem diameter (mm)	0.529*	0.317	0.542*					
Crude protein content (%)	0.811**	0.556*	0.621*	0.565*				
NDF (%)	0.105	0.35	0.06	0.512*	0.168			
ADF (%)	0.104	0.225	0.098	0.554*	0.157	0.718**		
Chlorophyll content (mg kg^−1^)	0.550*	0.801**	0.484	0.289	0.510*	0.266	0.169	
Content of soluble suger (mg kg^−1^)	0.420	− 0.850	0.338	0.205	0.567*	− 0.295	− 0.381	0.125

*Significant correlation was found at the 0.05 level (bilateral), **significant correlation was found at the 0.01 level (bilateral)

### Comprehensive evaluation

Because each treatment had different effects on different indicators, we could not comprehensively assess the optimal inoculation treatment with any single indicator. Thus, we used the following nine indicators of alfalfa for a comprehensive evaluation: aboveground biomass, infection rate, plant height, stem diameter, crude protein, NDF, ADF, chlorophyll content and soluble sugar content. We comprehensively evaluated the growth characteristics, nutritional quality, chlorophyll content and soluble sugar content of alfalfa under different inoculation treatments (Table 5). The results showed that aboveground biomass, infection rate, plant height, stem thickness, crude protein content, chlorophyll content and soluble sugar content were good indicators. In contrast, NDF and ADF content were negative indicators. The membership function values of the nine indexes were sorted by comprehensive value. Higher average values indicated higher comprehensive values, and lower average values indicated lower comprehensive values. According to the comprehensive ranking of alfalfa production indicators under different inoculation treatments, the top three inoculation treatments were as follows: J_3_P_2_ > J_1_P_2_ > J_3_P_1_.

**Table 5:** Comprehensive evaluation of various Indicators of alfalfa under different treatments

Index	Aboveground biomass (g pot^−1^)	Infection rate (%)	Plant height (cm)	Stem diameter (mm)	Crude protein (%)	NDF (%)	ADF (%)	Chlorophyll content (mg kg^−1^)	Content of soluble suger (mg kg^−1^)	Average	rank
J_0_P_0_	0.000	0.000	0.000	0.000	0.000	0.400	0.154	0.174	0.122	0.094	16
J_0_P_1_	0.440	0.313	0.140	0.748	0.068	0.840	0.673	0.381	0.381	0.443	9
J_0_P_2_	0.556	0.183	0.498	0.725	0.137	0.481	1.000	0.478	0.334	0.488	7
J_0_P_3_	0.336	0.003	0.241	0.557	0.200	1.000	0.742	0.385	0.000	0.385	13
J_1_P_0_	0.500	0.499	0.339	0.275	0.268	0.197	0.342	0.628	0.288	0.370	14
J_1_P_1_	1.000	0.728	0.442	0.718	0.337	0.654	0.534	0.765	0.438	0.624	4
J_1_P_2_	0.893	0.769	0.758	0.595	0.400	0.882	0.652	0.789	0.508	0.694	2
J_1_P_3_	0.842	0.044	0.545	0.618	0.468	0.289	0.357	0.101	0.440	0.412	12
J_2_P_0_	0.478	0.047	0.369	0.015	0.532	0.000	0.183	0.000	0.238	0.207	15
J_2_P_1_	0.609	0.275	0.483	0.389	0.600	0.602	0.205	0.397	0.782	0.482	8
J_2_P_2_	0.695	0.312	0.459	0.718	0.668	0.338	0.326	0.397	1.000	0.546	5
J_2_P_3_	0.792	0.226	0.317	0.321	0.732	0.176	0.000	0.514	0.725	0.423	10
J_3_P_0_	0.502	0.679	0.264	0.214	0.800	0.228	0.193	0.704	0.181	0.418	11
J_3_P_1_	0.884	1.000	0.476	0.328	0.868	0.749	0.630	1.000	0.251	0.687	3
J_3_P_2_	0.895	0.861	1.000	1.000	0.932	0.567	0.364	0.866	0.378	0.763	1
J_3_P_3_	0.644	0.041	0.572	0.305	1.000	0.043	0.069	0.802	0.933	0.490	6

P_0_, P_1_, P_2_, and P_3_ represent 0 mg kg^−1^, 50 mg kg^−1^, 100 mg kg^−1^, and 150 mg P kg^−1^, respectively. J_0_, J_1_, J_2_, and J_3_ represent CK, Fm, Bm, and Fm × Bm, respectively

## Discussion

### Effects of vaccination and phosphorus application on alfalfa root characteristics and aboveground growth

Arbuscular mycorrhizal fungi (AMF) are ubiquitous plant-fungal symbionts in nature (Wyss et al. 2016). AMF increase the absorption of mineral nutrients (especially phosphorus), increase the rate of photosynthesis, promote plant growth and improve plant quality (Delavaux et al. 2017). In our study, both the single bacteria injection and the mixed bacteria injection significantly promoted the growth of alfalfa compared to the treatment without bacteria (Table 1). AMF increase the uptake area of plant roots by forming an extensive hyphae network in the soil, thereby increasing the uptake of nutrients by roots and promoting plant growth (Wahid et al. 2019). Simultaneously, phosphorus-dissolving bacteria convert insoluble phosphorus in the soil into available phosphorus that can be absorbed and utilized by plants, thereby improving plant phosphorus nutrition and promoting plant growth and development (Barea 1991). Coinoculation with phosphate-solubilizing bacteria and phosphorus significantly increases wheat biomass in Alberta, Canada (Zhang et al. 2013). With regard to wheat research, different plant growth-promoting characteristics of PGPR bacteria and PSB are correlated to enhanced plant growth (Gianfreda 2015). Other studies have shown that after inoculation with AMF, the accumulation of regulatory substances is significantly improved. At the same time, the wilting rate of plants is reduced, and the efficiency of life metabolic activities is enhanced. Moreover, the aboveground biomass is increased (Eulenstein et al. 2016). Studies have demonstrated that after AMF infect plants, hyphae extend through the rhizosphere nutrient-deficient area to the adjacent soil, expanding the nutrient absorption range of the underground part (Yadav et al. 2017). Moreover, AMF improve the uptake rate of nutrients by host plants by increasing root uptake area (such as nitrogen and phosphorus), increasing soil spatial utilization rate and promoting the fluidity of mineral elements for better utilization (Delavaux et al. 2017). The growth-promoting effect of vaccination on alfalfa was evident in the present study.

The essential nutrient element for plant growth is phosphorus, which plays a critical role in plant metabolism and is also one of the leading nutrient elements affecting the improvement of crop yield. Phosphorus participates in various metabolic processes in plants in a variety of ways, thereby affecting plant physiology and morphology (Shen et al. 2011). In the present study, the mycorrhizae infection rate of alfalfa increased first and then decreased with the increase of phosphorus application (Fig. 2). Studies have shown that root exudates reduce soil pH and promote the conversion of some insoluble phosphorus to soluble phosphorus, thereby improving plant uptake of phosphorus (Wang et al. 2012). In addition, excessive application of phosphorus fertilizer (more than 750 kg ha^−1^) decreases the activity of enzymes in organic acid metabolism and the secretion of organic acids, thus reducing the activation and diffusion of phosphorus (Wang et al. 2014). The same trend was found in the growth of alfalfa shoots (Table 1). The lack of phosphorus hinders plant ATP synthesis, which affects plant photosynthesis and photophosphorylation (Chen et al. 2017). Phosphorus application improves alfalfa yield by increasing plant height (Zhang et al. 2020b). Another method for plants to gain phosphorus is AMF. Microorganisms around the extra root hyphae may affect the utilization of organophosphorus by AMF (Itoo and Reshi 2013). Thus, it is a crucial way to improve the efficiency of a plant using organic phosphorus to give full play to the interaction effect of AMF and PSB (Barea 1991).

### Effects of vaccination and phosphorus application on nutritional quality, chlorophyll content and soluble sugar content of alfalfa

The important indexes for evaluating the nutritional quality of alfalfa include the CP, ADF and NDF contents (Larson and Mayland 2007). The present study suggested that inoculation with AMF and PSB significantly promoted the nutritious quality of alfalfa under different phosphorus application conditions (Fig. 3, Table 2). Phosphorus nutrition plays an important role in alfalfa production. With phosphorus deficiency, plants improve their ability to absorb phosphorus by promoting root development, thereby affecting their nutritional quality (Narang and Altamann 2001). Simultaneous inoculation of PSB and AMF is beneficial to the phosphorus cycle in soil by improving the diversity of soil microorganisms (Hu et al. 2013), producing metabolites beneficial to plants and promoting their growth and nutrient absorption as well as stimulating and regulating plant growth to varying degrees. Inoculation of AMF on tomatoes improves their nutritional quality, and PGPR inoculation on white lupin grains enhances their nutritional quality (Miranda et al. 2015; Ferchichi et al. 2019). Phosphorus application increases the crude protein content in plants and regulates the content of washing and picking, thereby affecting their nutritional quality (Zhang et al. 2020b). When phosphorus application exceeds the maximum phosphorus uptake by alfalfa, the hay yield and phosphorus content of alfalfa plants decrease, which harms plant growth and development (Zhang et al. 2020a).

The chlorophyll content and leaf soluble sugar content can be used as essential indicators of nutrients for efficient growth of alfalfa (Elfanssi et al. 2018). The soluble sugar content, soluble protein content and nitrate reductase activity in tomato leaves inoculated with AMF are all increased compared to those without inoculation, and the mycorrhizae infection rate is positively correlated to the dry matter and soluble sugar content of plant stems and roots (Abbasifar et al. 2019). In our study, the chlorophyll content and leaf soluble sugar content of alfalfa under inoculation treatment were significantly greater than those under the noninoculation treatment (Table 3). Studies have shown that AMF significantly promote the uptake of mineral nutrients, especially phosphorus, by roots after symbiosis with roots and that AMF increase phosphorus content in plants even when the soil temperature decreases, plant growth is inhibited and phosphorus uptake is inhibited (Karasawa et al. 2012). At the same time, inoculation with AMF increases the relative moisture content of plant leaves, xylem pressure potential, stomatal conductance and photosynthetic rate of leaves, thereby increasing chlorophyll content (Caravaca et al. 2003). The coinoculation of AMF and PSB increases soil phosphatase activity in soil, thereby increasing the soluble sugar content of leaves. Moreover, the accumulation of soluble sugar content in leaves reduces the cell water potential and increases the intracellular and extracellular osmotic potential difference so that the external water is conducive for expansion into the cells, thereby maintaining plant growth (Barea 1991).

In conclusion, the present study showed that the appropriate phosphorus application and inoculation with AMF and PSB significantly increased the mycorrhizae infection rate, aboveground biomass, chlorophyll content in leaves and soluble sugar content of alfalfa. In contrast, excessive phosphorus application inhibited the increase in the above indicators. The CP, NDF and ADF contents of alfalfa showed a tendency to increase first and then decrease with increasing phosphorus application under the same bacterial treatments. Considering all factors, the best combination treatment was 100 mg kg^−1^ phosphorus application and coinoculation of Fm and Bm, which promoted the growth of mycorrhiza, promoted the growth of alfalfa shoots and improved the nutritional quality. Further investigation on the interaction between AMF and PSB will provide a theoretical basis for practical production and application.

## Data Availability

Please contact the authors for all requests.

## Abbreviations

AMF: Arbuscular mycorrhizal fungi
PSB: Phosphate-solubilizing bacteria
CP: Crude protein
ADF: Acid detergent fiber
NDF: Neutral detergent fiber
